# FMDNet: Spatial‐frequency feature routing for low‐dose CT denoising

**DOI:** 10.1002/acm2.70656

**Published:** 2026-06-15

**Authors:** Yujie Yao, Yunsen Liang, Wen Xiao, Zibo Zhou, Yaoxue Xu, Yonghao Pan, Xiao Xia

**Affiliations:** ^1^ School of Information Engineering Sichuan Agricultural University Ya'an Sichuan China; ^2^ College of Science Sichuan Agricultural University Ya'an Sichuan China; ^3^ Department of Radiology, Clinical Skills Center Ya'an People's Hospital Ya'an Sichuan China

**Keywords:** deep learning, image denoising, low‐dose computed tomography (LDCT)

## Abstract

**Background:**

Low‐dose computed tomography (LDCT) is widely used to reduce radiation exposure, but the reduced photon budget amplifies quantum noise and can introduce structured artifacts that obscure subtle boundaries and textures. Many deep learning denoisers process features in a single stream, which may encourage either over‐smoothing of weak anatomical edges or unstable texture synthesis.

**Purpose:**

To develop an LDCT denoising network that explicitly routes coarse structural content and fine details through dedicated pathways, aiming to suppress noise while preserving anatomically meaningful high‐frequency information.

**Methods:**

We propose FMDNet, a frequency‐aware encoder‐decoder equipped with an explicit coarse/detail routing block. A fixed low‐pass operator produces a coarse component, while the corresponding detail component is formed as an explicit residual (high = x—low). Each component is refined with depthwise operators and fused by a learned channel gate with channel attention. We evaluate FMDNet on the AAPM Mayo Clinic LDCT dataset and the LoDoPaB‐CT benchmark under a reproducible HU‐windowed protocol and supplement PSNR, SSIM, and RMSE with texture‐ and structure‐oriented analyses including HU line profiles and noise power spectrum (NPS), together with volume‐level paired tests and bootstrap confidence intervals on LoDoPaB‐CT.

**Results:**

Across both benchmarks, FMDNet achieves competitive quantitative fidelity and shows favorable results on complementary structure‐/texture‐oriented analyses. On Mayo, it improves mean PSNR, SSIM, and RMSE relative to strong learned baselines. On LoDoPaB‐CT, volume‐level analysis across 28 validation volumes confirms consistent improvements over Uformer with paired tests and bootstrap confidence intervals. Additional HU‐profile and NPS analyses provide complementary evidence of improved texture preservation and local structural fidelity relative to comparative methods.

**Conclusions:**

Explicitly separating coarse and detail residual components in feature space provides a practical inductive bias for LDCT denoising. When combined with gated fusion and multi‐scale supervision, this strategy improves quantitative fidelity and preserves fine structures without relying on adversarial texture synthesis; clinical diagnostic impact should be validated by reader studies.

## 1 INTRODUCTION

Computed tomography (CT) plays a central role in diagnostic imaging, yet radiation exposure remains a concern, particularly for screening and longitudinal follow‐up. Dose reduction strategies lower patient risk but increase quantum mottle noise and can alter the appearance of subtle lesions and weak boundaries.^1^

Classical approaches include analytical reconstruction and iterative, model‐based reconstruction techniques,^2^, ^3^, ^4^, ^5^ as well as image‐domain denoising methods applied post‐reconstruction.^6^, ^7^, ^8^, ^9^, ^10^, ^11^ While often effective, these approaches may be computationally demanding and tightly coupled to scanner‐specific physics and implementations.

Deep learning has emerged as a practical alternative by learning a mapping from noisy LDCT to normal‐dose CT (NDCT) from paired or self‐supervised data. CNN‐based methods are stable and efficient but tend to trade texture fidelity for noise suppression, leading to over‐smoothing of low‐contrast structures.^12^, ^13^ GAN‐based approaches can restore perceptual sharpness but may hallucinate textures that are not fully supported by the data, which is undesirable in medical imaging.^14^, ^15^, ^16^, ^17^, ^18^, ^19^, ^20^ Transformer‐style models improve long‐range context modeling but still commonly apply uniform processing over feature maps and may average away faint edges.^21^, ^22^

A recurring limitation across these families is the absence of an explicit mechanism to treat coarse structures and fine detail/noise differently. In CT images, low‐frequency content primarily encodes slowly varying anatomy and organ interiors, whereas high‐frequency components carry edges and small structures but also include noise. Processing everything with a single stream forces the network to implicitly disentangle these components, often resulting in conservative blur or aggressive, potentially unstable sharpening.

Inspired by multi‐scale decompositions such as Laplacian pyramids and split‐transform‐merge designs, we introduce an explicit, interpretable coarse/detail routing prior inside a compact restoration block.^23^ Rather than performing explicit frequency transforms, we construct the decomposition in the spatial domain using a fixed low‐pass operator and an explicit residual high‐pass component. This simple construction makes it difficult for the detail branch to replicate low‐frequency content and encourages targeted refinement where it is most needed.

## 2 RELATED WORK

CNN‐based LDCT denoising remains a strong baseline due to locality, parameter efficiency, and training stability.^12^, ^13^, ^24^ However, purely convolutional approaches may oversmooth weak edges when optimized primarily for pixel‐wise losses.

GAN‐based denoisers can mitigate perceptual blur and enhance local contrast, but adversarial objectives may encourage texture synthesis that is not guaranteed to be anatomically faithful.^14^, ^15^, ^16^, ^17^, ^18^, ^19^, ^20^, ^25^ This risk motivates conservative objectives and complementary analyses beyond single scalar metrics.

Transformer‐style restoration models capture long‐range dependencies via attention, with U‐shaped designs (e.g., Uformer) widely used for image restoration.^21^, ^22^ Recent state‐space models such as Mamba provide an alternative route to global modeling with linear complexity, but converting 2D feature maps to sequences can weaken spatial inductive biases.^26^

Frequency‐inspired and multi‐scale methods have a long history in image processing. Laplacian pyramids provide an interpretable decomposition across image scales and are often used as explicit multi‐resolution representations or supervisory priors.^23^ Split‐transform‐merge strategies such as Octave Convolution route predefined channel subsets at different spatial resolutions.^27^ Our approach is related in motivation but differs in mechanism. Unlike Laplacian pyramids and wavelet‐based decompositions that operate at image or explicit multi‐scale levels, our routing is applied within feature space at every block and is integrated into end‐to‐end optimization. More importantly, the detail branch is not an independent pathway but is explicitly constrained as a residual to the low‐pass component, which enforces complementarity and reduces low‐frequency leakage, an effect not guaranteed in existing frequency‐ or channel‐splitting methods.

Our contributions are threefold: (i) we propose an explicit coarse/detail routing block based on fixed low‐pass filtering and residual formation; (ii) we integrate the block into a four‐stage encoder‐decoder with gated fusion and efficient channel attention; and (iii) we report HU‐windowed evaluation on Mayo and LoDoPaB‐CT with quantitative metrics, ROI visuals, ablations, and texture‐preservation analyses, including HU profiles and the noise power spectrum (NPS), as complementary evidence rather than direct clinical validation.

Recent CT‐specific work has also explored hybrid transform‐learning and generative formulations. For example, Katta et al. combined shearlet‐domain decomposition with a method‐noise CNN, while Aneesh et al. studied a CT denoising framework based on autoencoder and GAN components.^28^, ^29^ In addition, a recent review of CT noise‐reduction methods emphasized that model validation, generalizability, and clinically relevant image‐quality assessment are as important as raw metric gains in CT denoising studies.^30^

To provide broader context, prior studies have explored CT physics and radiation dose considerations.^31^, ^32^, ^33^, ^34^ Classical and model‐based reconstruction methods have also been extensively developed.^3^, ^4^, ^5^, ^35^, ^36^ Furthermore, learning‐based image restoration approaches remain active areas of research.^1^, ^37^, ^38^, ^39^, ^40^, ^41^, ^42^, ^43^, ^44^

## 3 METHODS

### 3.1 Network overview

**FIGURE 1:**
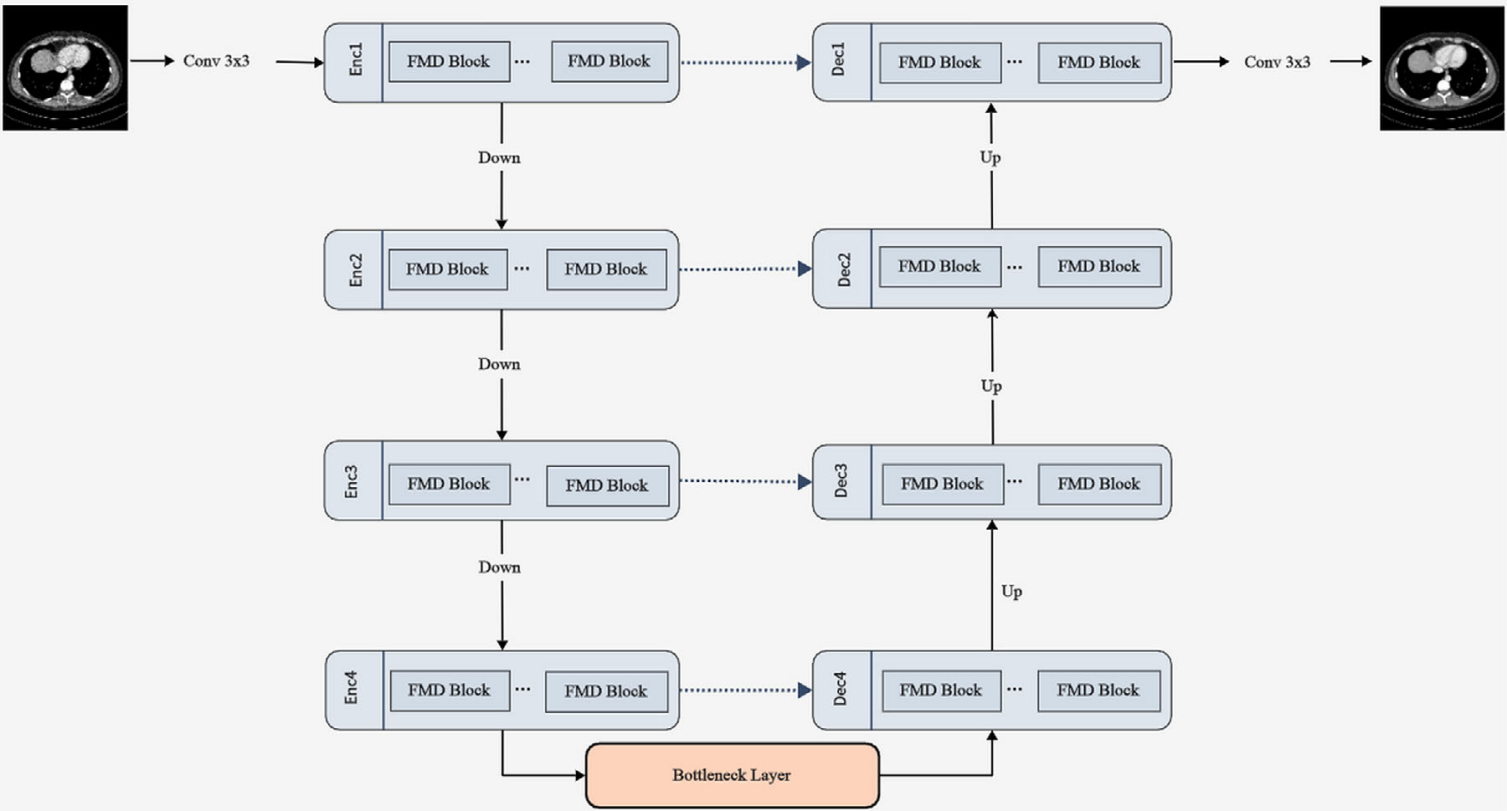
Overall architecture of FMDNet. A four‐stage encoder–decoder backbone uses FMDBlocks at each resolution, with skip connections linking encoder and decoder features to preserve spatial detail.

**FIGURE 2:**
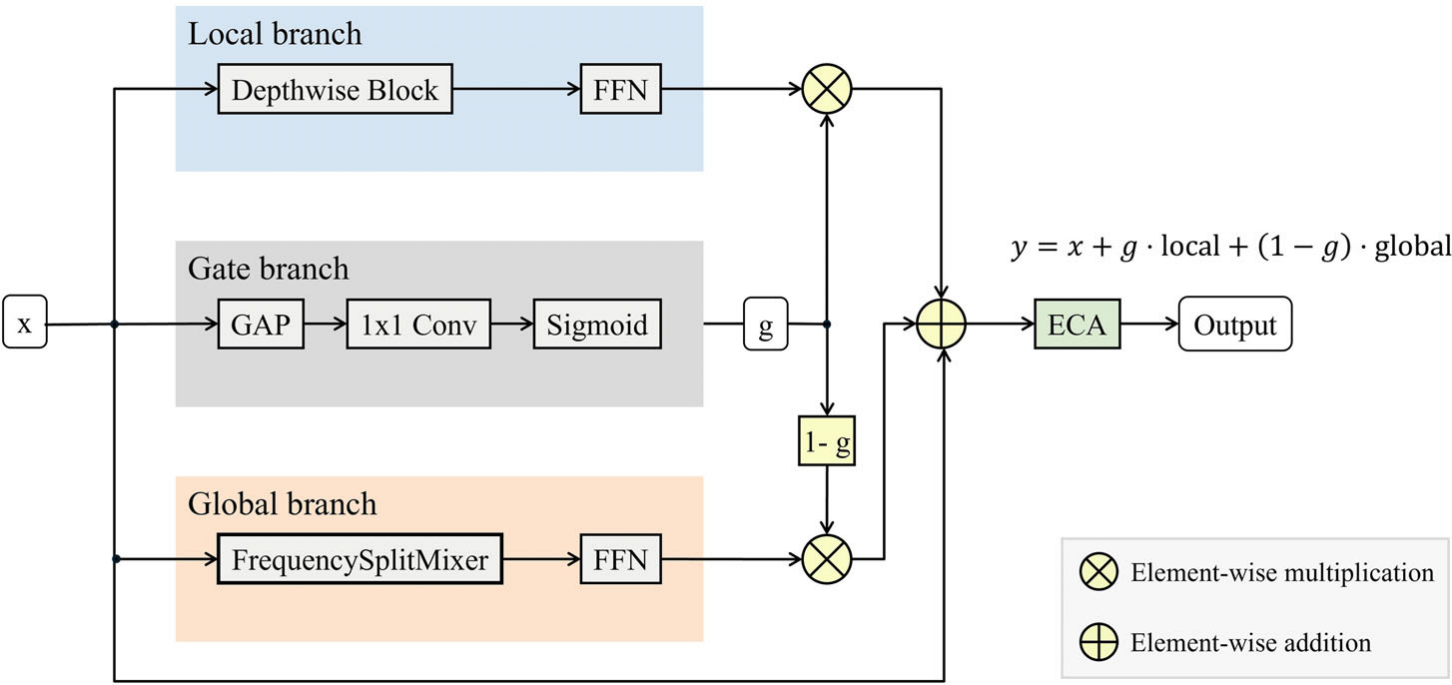
FMDBlock design. The block combines a local depthwise stream and a global frequency‐routing stream (FreqSplitMixer), fused by a learned gate and followed by efficient channel attention (ECA) for channel reweighting.

FMDNet follows a four‐stage encoder‐decoder backbone with U‐Net‐style skip connections (Figure 1).^12^ A 3×3 stem convolution maps the LDCT input to feature space. The encoder progressively downsamples feature maps while increasing channel capacity, and the decoder mirrors this process with upsampling and convolutional refinement. Skip connections convey spatial detail and facilitate optimization. The main restoration capacity is provided by repeated routed restoration blocks, each combining explicit coarse/detail routing with pointwise channel mixing. An FMDBlock integrates a local depthwise pathway and a global frequency‐routing pathway (FreqSplitMixer), fused by a learned gate and followed by efficient channel attention (Figure 2).

Given a feature tensor $x\in R^{C\times H\times W}$, we form a deterministic two‐stream decomposition. The coarse component is obtained by a fixed low‐pass operator G(·), and the detail component is formed as an explicit residual, x—G(x). Thus, before branch‐specific refinement, the two streams are complementary under the chosen low‐pass operator and sum back to the original feature tensor. This construction encourages the detail stream to focus on edges, textures, and residual noise while the coarse stream stabilizes slowly varying anatomical structures. In contrast, generic channel‐splitting approaches do not impose this complementary relation and allow their nominal high‐frequency path to retain unconstrained low‐frequency content.

(1) $$\;x_{\mathrm{low}}=G\left(x\right),\;x_{\mathrm{high}}=x-x_{\mathrm{low}}$$

**FIGURE 3:**
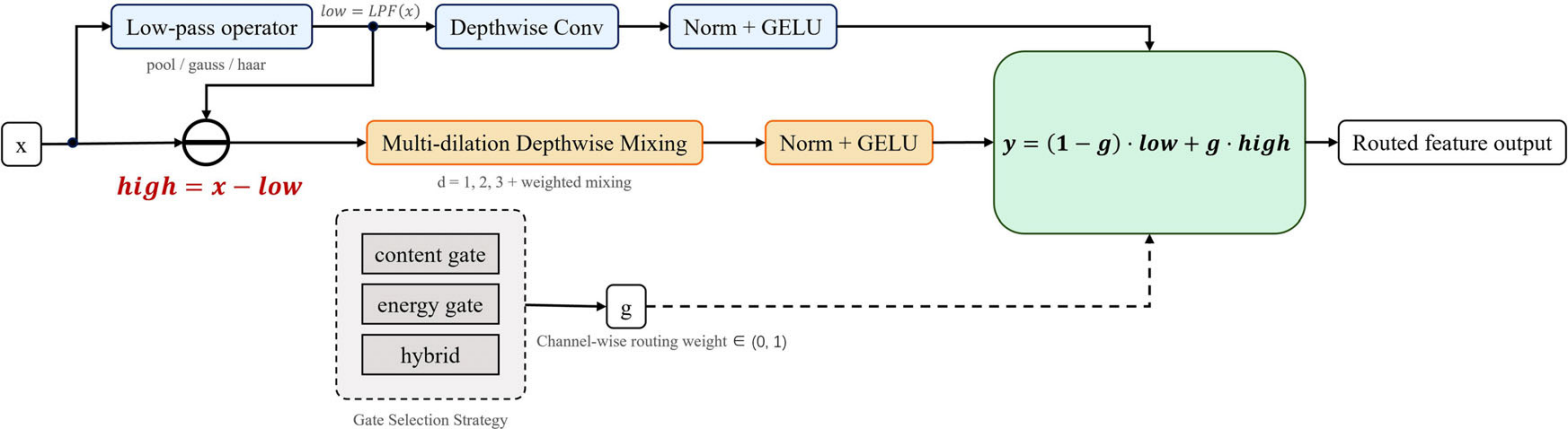
FrequencySplitMixer for coarse/detail routing. A fixed low‐pass filter extracts a low‐frequency component, and the high‐frequency component is formed as a residual (high = x − low). Each branch is refined by depthwise operators and fused using a learned gate.

In our default implementation, G(·) is realized by 2×2 average pooling followed by nearest‐neighbor upsampling, which is fast and hardware‐friendly. Alternative fixed low‐pass operators (e.g., Gaussian blur or Haar‐wavelet LL reconstruction) can be substituted for analysis without changing the rest of the network. Each stream is refined by lightweight depthwise operators: the coarse stream focuses on smoothing and stabilizing homogeneous regions, while the detail stream uses depthwise mixing (optionally with multiple dilations) to refine boundaries while suppressing residual noise. The detailed routing and refinement strategy is illustrated in Figure 3.

The two refined streams are fused by a learned channel gate computed from global average pooled features. Let σ denote a sigmoid activation and GAP(·) denote global average pooling; then the fused output is

(2) $$\begin{array}{cc} g=\sigma \left(\mathrm{Con}v_{1\times 1}\left(\mathrm{GAP}\left(x\right)\right)\right),\;y & =\left(1-g\right)\;⊙f_{\mathrm{low}}\left(x_{\mathrm{low}}\right)\\ & \;+g⊙f_{\mathrm{high}}\left(x_{\mathrm{high}}\right) \end{array}$$

### 3.2 Channel reweighting and pointwise mixing

After gated fusion, we apply efficient channel attention (ECA) to adaptively reweight channels using a lightweight local cross‐channel interaction, improving stability and reducing sensitivity to channel scaling.^45^ Finally, a pointwise feed‐forward network (1×1 convolutions with nonlinearity) mixes channels and produces the block output. Residual connections are used throughout to stabilize training in deeper settings.

### 3.3 Training objective

We optimize a composite objective that combines conservative pixel fidelity with structural and multi‐scale regularization:

(3) $$L=\lambda_{0}\;L_{0}+\lambda_{\mathrm{ssim}}L_{\mathrm{ssim}}+\lambda_{\mathrm{pyr}}L_{\mathrm{pyr}}$$

In our experiments, L0 denotes the dominant pixel‐fidelity term and is instantiated as either mean squared error (MSE) or mean absolute error (MAE, also referred to as L1 loss). SSIM loss encourages structural consistency.^46^ Laplacian pyramid regularization encourages agreement across scales and has been used widely in restoration.^23^

### 3.4 Experimental setup

#### 3.4.1 Datasets and splits

We evaluate FMDNet on two public benchmarks. (i) Mayo Clinic LDCT (AAPM Grand Challenge): 10 cases with paired low‐dose and normal‐dose reconstructions.^47^ We use the standard paired‐slice setup and report quantitative results on held‐out cases (L506 and L333), using the remaining cases for training. (ii) LoDoPaB‐CT (simulated low‐dose CT with ground‐truth reference images): we follow the official split (280 training volumes, 28 validation volumes) and report metrics on the full validation set.^48^ All data are publicly available and de‐identified; therefore institutional review board approval and informed consent were not required (see Table 1 for dataset statistics).

**TABLE 1:** Dataset statistics.

Dataset	Split	Cases/volumes	Slices
Mayo	all	10	5936
LoDoPaB‐CT	train	280	35820
LoDoPaB‐CT	validation	28	3522

*Note*: Slice counts are reported after pairing/alignment in the preprocessing pipeline.

#### 3.4.2 HU‐windowed evaluation protocol

To reduce ambiguity from intensity normalization, we evaluate under fixed HU windows. Predictions and references are converted to HU, clamped to a preset window, and linearly normalized to [0, 1] for computing PSNR and SSIM. RMSE is reported in HU under the same clamped window. For the results reported here, we use HU[−160, 240] for Mayo (soft tissue) and HU[−1000, 400] for LoDoPaB‐CT (lung).

#### 3.4.3 Baselines

We compare against representative CNN and Transformer baselines: RED‐CNN, DuGAN, Uformer, CTformer, and a Mamba‐style baseline (CTmamba).^12^, ^13^, ^20^, ^21^, ^22^, ^26^ All methods are evaluated at full resolution; when memory constraints apply, we use tiled inference following each baseline's recommended settings. Unless stated otherwise, model selection uses validation PSNR. For qualitative and classical‐reference comparisons, we additionally include a Wiener filter baseline (non‐learning; 5×5 neighborhood) in the quantitative tables and ROI figures.

#### 3.4.4 Implementation and training details

All experiments were implemented in PyTorch with CUDA 11.8 and run on a workstation equipped with Ubuntu 20.04.6 LTS and an NVIDIA GeForce RTX 4090 GPU (24 GB). For the proposed model, training uses 2D axial patches (128×128, stride 64), random flips, and random 90° rotations. Optimization uses AdamW with cosine learning‐rate scheduling and automatic mixed precision, and an exponential moving average (EMA; decay 0.999) of model weights is used for checkpoint selection.^49^, ^50^, ^51^, ^52^ The final FMDNet models for Mayo and LoDoPaB‐CT were trained for 320 and 140 epochs, respectively, with validation every 5 epochs. Baseline methods followed their own repository‐specific training settings and tiled inference configurations where required. The fixed HU windows described above are used for evaluation; training preprocessing followed each dataset's native normalized intensity representation.

Use of generative AI and large language models: During preparation of this manuscript, a large language model (ChatGPT, OpenAI) was used for language editing and clarity. The authors reviewed and verified the final content and take full responsibility for the work.

## 4 RESULTS

### 4.1 Quantitative evaluation

Tables 2 and 3 summarize quantitative results on Mayo (L506+L333) and LoDoPaB‐CT validation, respectively. We report mean ± standard deviation across slices for PSNR, SSIM, and RMSE(HU). Standard fidelity metrics do not fully capture clinically relevant texture characteristics and local structural fidelity. We therefore additionally report case‐/volume‐level aggregation and complementary texture‐oriented analyses using HU profiles and NPS.

**TABLE 2:** Quantitative results on Mayo (L506 and L333).

Model	PSNR	SSIM	RMSE(HU)
LDCT	23.4417 ± 1.8799	0.8149 ± 0.0461	27.5499 ± 5.9810
Wiener	26.4464 ± 1.7637	0.8323 ± 0.0418	19.4391 ± 3.9611
RED‐CNN	28.4775 ± 1.5875	0.8687 ± 0.0353	15.3307 ± 2.8935
DuGAN	28.5970 ± 1.5667	0.8613 ± 0.0379	15.1149 ± 2.8190
Uformer	28.7388 ± 1.5851	0.8694 ± 0.0364	14.8758 ± 2.8069
CTformer	28.5637 ± 1.5846	0.8642 ± 0.0377	15.1786 ± 2.8626
CTmamba	28.4769 ± 1.5878	0.8665 ± 0.0355	15.3317 ± 2.8910
**FMDNet (Proposed)**	**28.9618 ± 1.5891**	**0.8796 ± 0.0358**	**14.6379 ± 2.8091**

*Note*: Mayo evaluation uses a fixed soft‐tissue window (HU [−160, 240]). Values are mean ± SD across slices.

**TABLE 3:** Quantitative results on LoDoPaB‐CT validation.

Model	PSNR	SSIM	RMSE(HU)
LDCT	26.2531 ± 2.5251	0.6063 ± 0.1562	71.2996 ± 23.2759
Wiener	26.9344 ± 2.0092	0.6849 ± 0.1303	64.9269 ± 18.3422
RED‐CNN	30.7201 ± 2.8723	0.7727 ± 0.1385	43.4422 ± 19.0860
DuGAN	32.0911 ± 3.3295	0.7988 ± 0.1410	37.9148 ± 19.4747
Uformer	31.8790 ± 3.2414	0.7966 ± 0.1388	38.6721 ± 19.2709
CTformer	30.5774 ± 2.9179	0.7673 ± 0.1351	44.2278 ± 19.4761
CTmamba	29.3006 ± 2.5274	0.7345 ± 0.1295	50.3601 ± 18.6533
**FMDNet (Proposed)**	**32.4764 ± 3.2556**	**0.8108 ± 0.1396**	**35.2739 ± 19.2180**

*Note*: LoDoPaB‐CT evaluation uses a fixed lung window (HU [−1000, 400]). Values are mean ± SD across slices.

Because slices from the same volume are not fully independent, statistical testing at the slice level should be interpreted cautiously. We therefore emphasize effect sizes and consistency across slices. Case‐level metrics for the two Mayo test cases are additionally summarized in Table 4 to mitigate slice‐level dependence. For LoDoPaB‐CT, we further report volume‐level aggregation (one sample per volume), paired tests, and bootstrap 95% confidence intervals in Table 5.

**TABLE 4:** Case‐level quantitative results on Mayo under the soft‐tissue window (HU[−160, 240]).

Model	L506 PSNR (dB)	L506 SSIM	L506 RMSE (HU)	L333 PSNR (dB)	L333 SSIM	L333 RMSE (HU)
LDCT	24.469 ± 2.040	0.8445 ± 0.0399	24.637 ± 6.558	22.556 ± 1.140	0.7895 ± 0.0343	30.062 ± 3.988
Wiener	27.408 ± 1.910	0.8587 ± 0.0380	17.498 ± 4.339	25.617 ± 1.077	0.8096 ± 0.0298	21.113 ± 2.633
RED‐CNN	29.109 ± 1.821	0.8904 ± 0.0322	14.350 ± 3.358	27.935 ± 1.094	0.8500 ± 0.0260	16.174 ± 2.081
DuGAN	29.188 ± 1.797	0.8841 ± 0.0351	14.210 ± 3.269	28.088 ± 1.108	0.8416 ± 0.0280	15.894 ± 2.067
Uformer	29.340 ± 1.824	0.8914 ± 0.0338	13.974 ± 3.264	28.225 ± 1.113	0.8504 ± 0.0266	15.647 ± 2.048
CTformer	29.176 ± 1.811	0.8874 ± 0.0346	14.235 ± 3.303	28.037 ± 1.119	0.8442 ± 0.0274	15.990 ± 2.106
CTmamba	29.115 ± 1.821	0.8882 ± 0.0321	14.339 ± 3.353	27.932 ± 1.092	0.8477 ± 0.0264	16.178 ± 2.070
**Proposed**	**29.469 ± 1.819**	**0.8934 ± 0.0328**	**13.905 ± 3.248**	**28.340 ± 1.123**	**0.8568 ± 0.0263**	**15.322 ± 2.065**

**TABLE 5:** Volume‐level quantitative results on LoDoPaB‐CT validation (28 volumes).

Model	Volumes (n)	PSNR (mean ± SD)	SSIM (mean ± SD)	RMSE(HU) (mean ± SD)
LDCT	28	26.2448 ± 0.2242	0.6058 ± 0.0154	71.3865 ± 2.0585
Uformer	28	31.8484 ± 0.2909	0.7951 ± 0.0126	38.8173 ± 1.6916
**Proposed**	**28**	**32.1245 ± 0.2931**	**0.8184 ± 0.0129**	**36.5063 ± 1.7014**

*Note*: Volume‐level paired differences were computed as Proposed − Uformer for PSNR and SSIM, and as Uformer − Proposed for RMSE so that positive values consistently indicate improvement. The resulting differences were ΔPSNR = 0.2761 dB (bootstrap 95% CI [0.2082, 0.3579]), ΔSSIM = 0.0233 (bootstrap 95% CI [0.0166, 0.0304]), and ΔRMSE = 2.311 HU (bootstrap 95% CI [1.417, 3.224]). Paired two‐sided t‐tests across volumes indicated significant differences for all three metrics (all p < 0.001). We report these volume‐level analyses to mitigate slice‐level dependence and to complement slice‐wise significance testing.

***Mayo dataset: slice‐level quantitative results (soft‐tissue window)***

***LoDoPaB‐CT validation: slice‐level quantitative results (lung window)***

***Mayo: case‐level results (L506 and L333)***

***LoDoPaB‐CT: volume‐level statistics (n = 28 volumes)***

### 4.2 Visual comparisons

**FIGURE 4:**
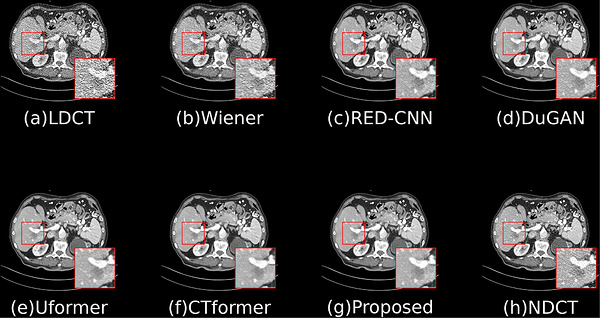
Representative Mayo Clinic LDCT ROI visual comparison. Panels show (a) LDCT, (b) Wiener filter, (c) RED‐CNN, (d) DuGAN, (e) Uformer, (f) CTformer, (g) Proposed, and (h) NDCT. Red boxes indicate the evaluated ROI with an enlarged inset.

**FIGURE 5:**
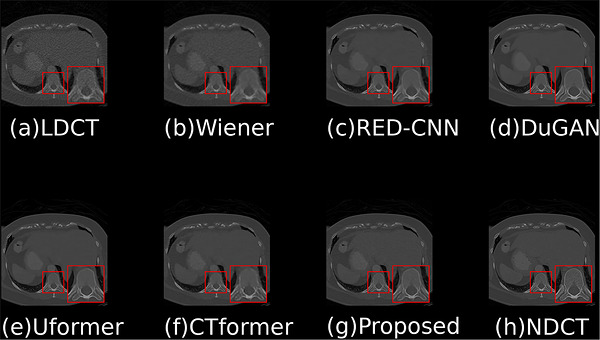
Representative LoDoPaB‐CT ROI visual comparison. Panels show (a) LDCT, (b) Wiener filter, (c) RED‐CNN, (d) DuGAN, (e) Uformer, (f) CTformer, (g) Proposed, and (h) the reference image (ground truth). Red boxes indicate the evaluated ROI with an enlarged inset.

To complement full‐slice metrics, we include ROI visual comparisons focusing on weak boundaries and fine texture patterns. For readability, Figures 4 and 5 show a subset of methods and additionally include a classical Wiener filter baseline (non‐learning; 5×5 neighborhood). Compared with CNN baselines, the proposed method tends to preserve boundary contrast with less diffuse blur; compared with GAN baselines, it avoids overly crisp textures that may deviate from the reference.

### 4.3 Ablation study

**FIGURE 6:**
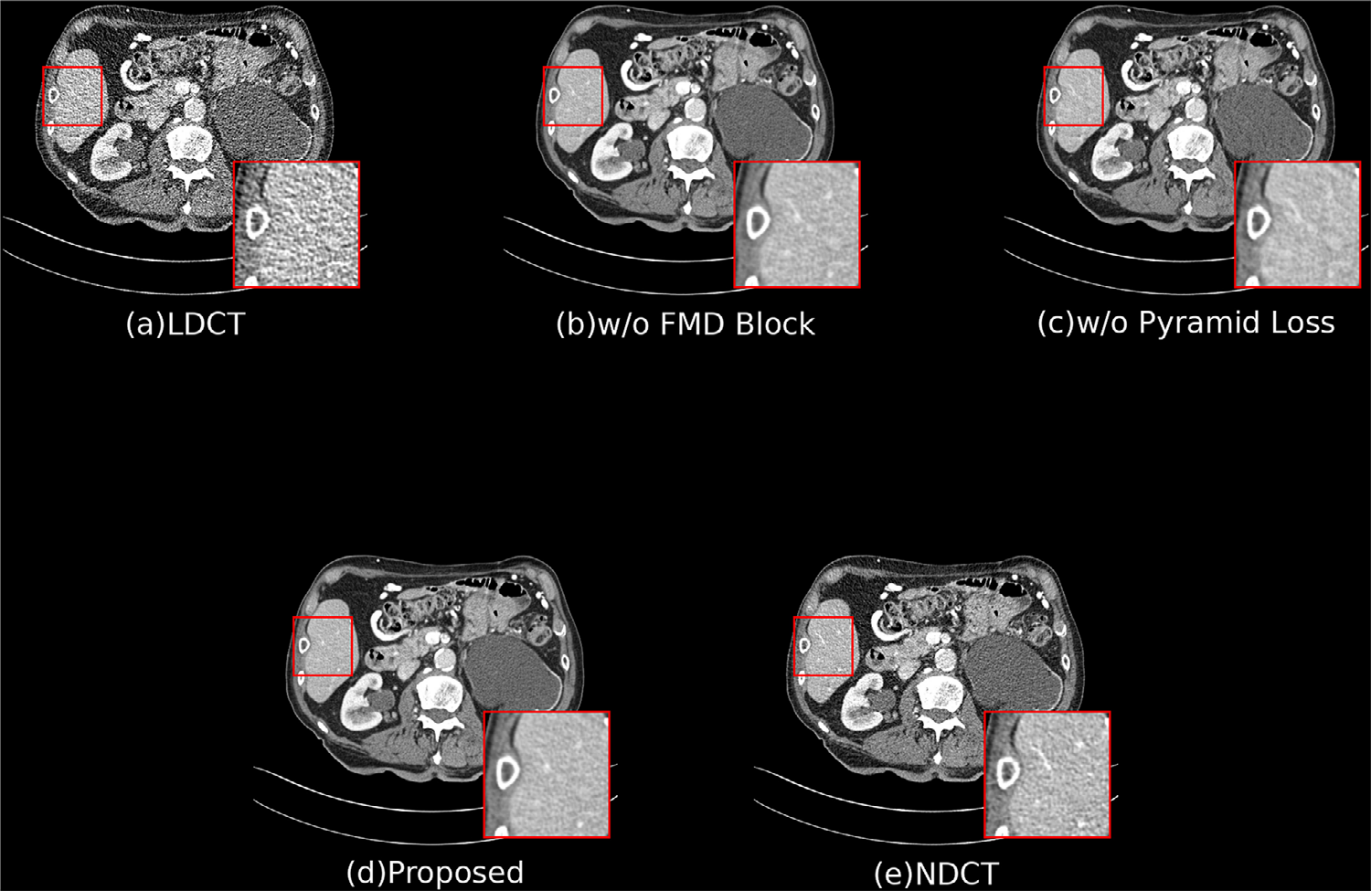
ROI ablation visualization on Mayo. Comparisons include the input LDCT, variants without the FMDBlock or pyramid loss, the proposed model, and the NDCT reference.

We perform ablations on Mayo to isolate the impact of the routed block and auxiliary losses. Table 6 reports mean ± SD across slices for a held‐out case (L506). Removing the routed block reduces PSNR/SSIM and increases RMSE, supporting the role of explicit coarse/detail routing. Removing the pyramid regularizer slightly degrades performance, consistent with a modest multi‐scale benefit. Visual comparisons of these ablations on the Mayo dataset are provided in Figure 6.

**TABLE 6:** Ablation results on Mayo (L506).

Ablation	PSNR	SSIM	RMSE(HU)
LDCT	24.4688 ± 2.0397	0.8445 ± 0.0399	24.6370 ± 6.5580
w/o routed block	29.2190 ± 1.8095	0.8902 ± 0.0325	14.0932 ± 3.2669
w/o pyramid loss	29.0546 ± 1.8572	0.8891 ± 0.0333	14.4552 ± 3.4724
**FMDNet (Proposed)**	**29.2973 ± 1.8134**	**0.8942 ± 0.0326**	**14.0348 ± 3.2665**

*Note*: Ablations are evaluated on Mayo case L506. Values are mean ± SD across slices.

***Mayo ablation study (L506)***

### 4.4 Texture‐ and structure‐preservation analyses

**FIGURE 7:**
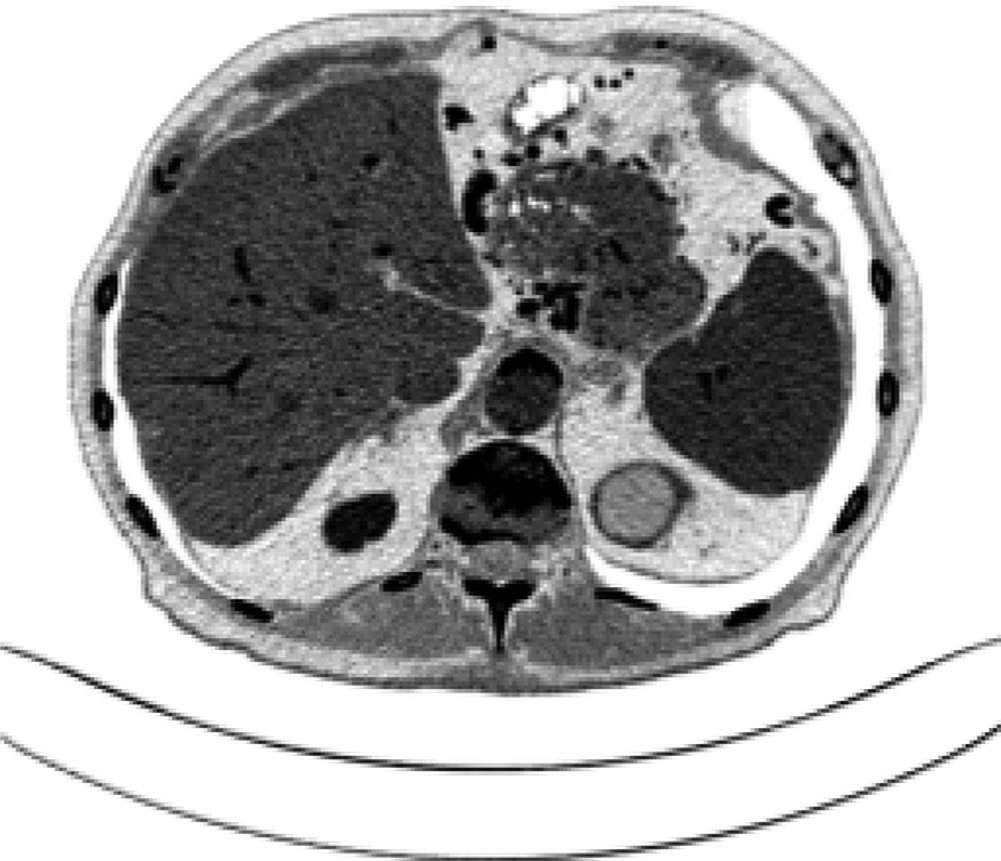
Low‐frequency (coarse) feature response from the FrequencySplitMixer (Mayo L506, slice 068). This panel represents an internal feature‐map summary for mechanism illustration rather than a reconstructed CT image.

**FIGURE 8:**
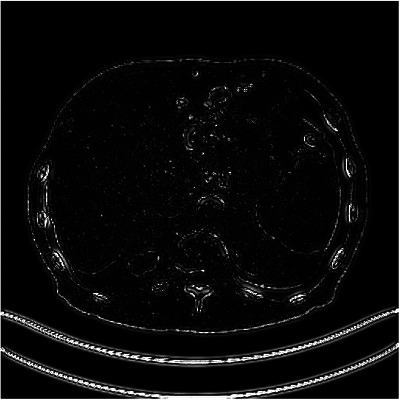
High‐frequency (detail) feature response from the FrequencySplitMixer (Mayo L506, slice 068). Similar visualization setup as Figure 7.

**FIGURE 9:**
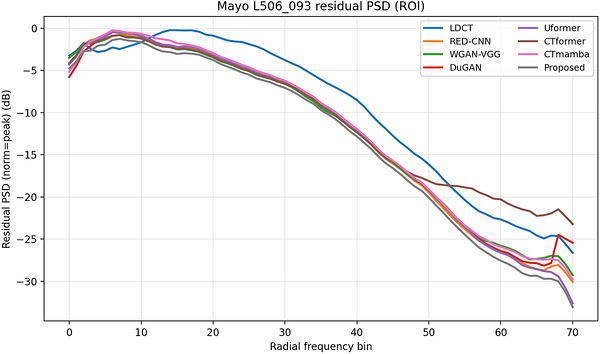
Residual power spectral density (PSD) on a representative Mayo ROI; residual is defined as reference minus output.

To intuitively illustrate the proposed routing mechanism, Figures 7 and 8 visualize the internal feature responses of a trained FMDNet using a representative Mayo slice (case L506, slice 068). Specifically, Figure 7 shows the coarse (low‐frequency) representation, while Figure 8 displays the detail‐oriented (high‐frequency) response extracted from the FrequencySplitMixer. To further assess local structural fidelity and texture characteristics, we provide complementary analyses using HU line profiles and NPS, alongside residual power spectral density (PSD) (Figure 9).

**FIGURE 10:**
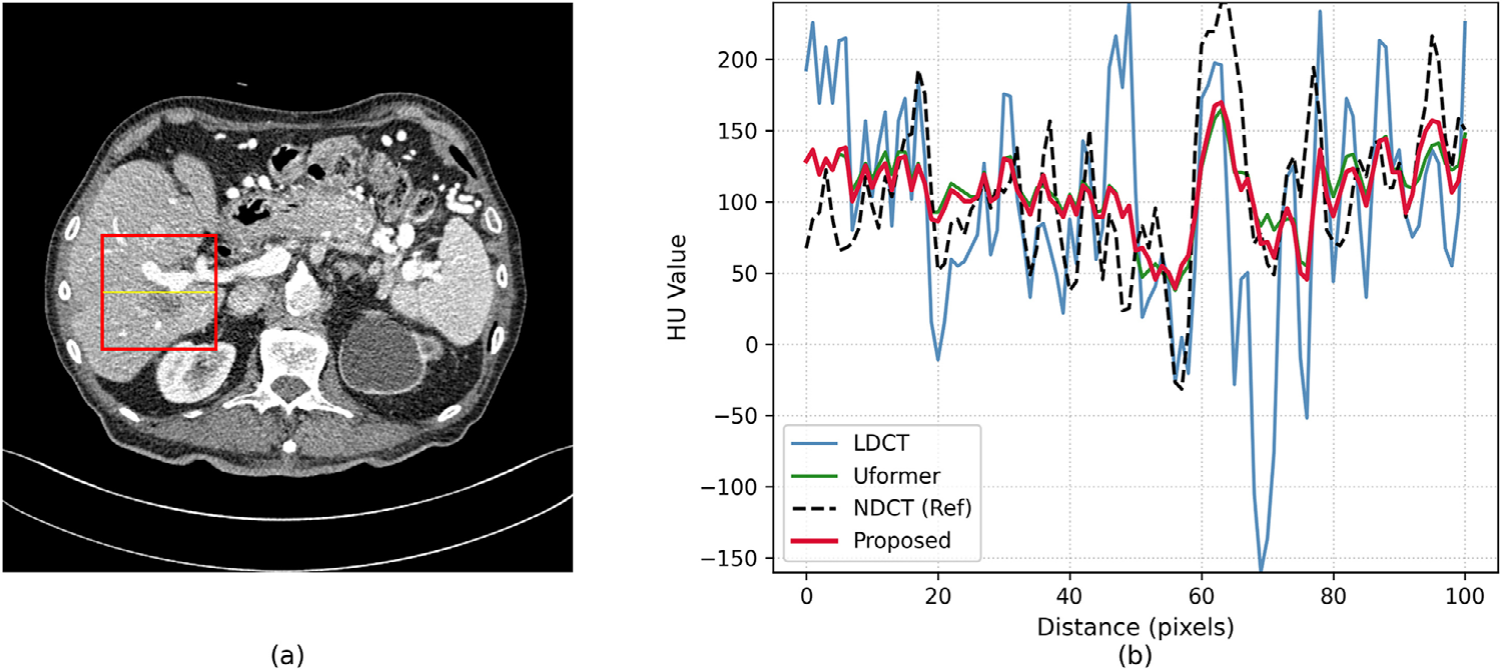
HU line‐profile and NPS ROI on Mayo case L506. (a) CT slice with the profile sampling line (yellow) and the 100×100 pixel NPS calculation ROI (red box). (b) HU line‐profile comparison showing the proposed method tracks the NDCT reference more closely than Uformer, preserving boundaries while suppressing noise.

**FIGURE 11:**
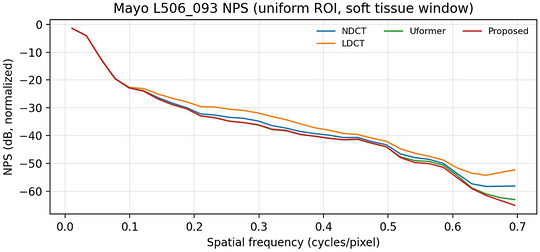
Noise power spectrum (NPS) on Mayo (case L506). Calculated from the 100×100 pixel ROI (red box in Figure 10a). Curves show normalized, radially averaged 2D NPS in dB.

Line‐profile and noise analysis. To complement the visual ROIs, Figure 10a marks a representative soft‐tissue boundary with a yellow line, and the corresponding HU values are plotted in Figure 10b, where a closer alignment with the reference (NDCT) curve indicates higher structural fidelity. The quantitative comparison confirms that in most regions along the profile, our proposed method tracks the reference more closely and preserves boundary transitions more faithfully than strong baselines like Uformer, which exhibits noticeable over‐smoothing. By explicitly routing coarse and detail features, our approach successfully reduces noise oscillations present in the LDCT while maintaining this high fidelity. Furthermore, to ensure transparency in noise‐texture assessment, a uniform 100×100 pixel ROI (red box, Figure 10a) was selected for NPS calculation. The 2D NPS was estimated via the squared magnitude of the 2D Fourier transform of the ROI. As shown in the radially averaged 1D NPS curves (Figure 11), our method reduces noise magnitude while maintaining a spectral shape close to the reference.

Figure 11 shows that the proposed method reduces noise magnitude while maintaining a spectral shape close to the reference compared with LDCT and a strong baseline.

***Noise‐texture analysis: noise power spectrum (NPS)***

## 5 DISCUSSION

FMDNet is designed around a simple premise: treating coarse structure and detail residuals as different signals can reduce the burden on downstream layers to disentangle noise from anatomy. The fixed low‐pass and explicit residual formation provide an interpretable decomposition and a conservative inductive bias that discourages the detail branch from reproducing low‐frequency content.

Empirically, the proposed routing improves quantitative fidelity on Mayo and produces strong results on LoDoPaB‐CT. Qualitative ROIs suggest that the model preserves weak edges more reliably than purely convolutional baselines while avoiding overly sharp textures sometimes associated with adversarial training. This constrained complementarity is particularly relevant for LDCT denoising, where high‐frequency components contain both anatomical edges and noise, making unconstrained detail enhancement prone to either blur or unstable sharpening. This interpretation is supported by complementary structure and texture analyses: the HU line profiles (Figure 10) show boundary transitions close to the reference with reduced oscillatory noise, and the NPS analysis (Figure 11) indicates a spectral shape closer to the reference than comparative methods.

An important caveat is that LoDoPaB‐CT is based on simulated low‐dose measurements and a controlled reconstruction pipeline. Its scale and exact references make it valuable for benchmarking, but the resulting noise statistics and texture may not fully reproduce vendor‐specific clinical LDCT, especially after scanner‐dependent preprocessing, reconstruction kernels, and iterative reconstruction. Accordingly, the LoDoPaB results should be interpreted as evidence under a standardized simulated low‐dose model rather than as direct proof of immediate clinical generalization. Simulation‐to‐clinical transfer may be possible when acquisition and noise characteristics are reasonably matched, but this remains task‐ and protocol‐dependent. The Mayo experiments partly complement this by using paired clinical reconstructions, yet broader external validation on real low‐dose acquisitions remains necessary.

Several limitations remain. First, although we include structure‐ and texture‐preservation proxies (HU line profiles and NPS) in addition to fidelity metrics, clinical diagnostic reliability still requires reader studies and task‐based endpoints.^53^ Second, domain shifts across scanner vendors, reconstruction kernels, and dose levels can change noise texture and frequency content. Potential mitigation strategies include scanner‐aware fine‐tuning, domain adaptation, self‐supervised refinement on unlabeled target‐domain LDCT data, and test‐time adaptation; however, such approaches were outside the scope of the present study. Finally, like all learning‐based denoisers, the method can produce atypical failures on out‐of‐distribution inputs; conservative operating points and quality‐control checks are advisable in safety‐critical workflows.

## 6 CONCLUSION

We presented FMDNet, an LDCT denoiser that explicitly routes coarse and detail residual components in feature space using a fixed low‐pass operator and residual formation. Combined with gated fusion and lightweight attention, this design improves quantitative fidelity and preserves fine structures without relying on adversarial objectives. Future work will extend validation across broader acquisition conditions and include reader studies to assess task‐level diagnostic impact.

## AUTHOR CONTRIBUTIONS

**Yujie Yao**: writing—original draft; methodology; validation; conceptualization; data curation. **Yunsen Liang**: writing—original draft; software. **Wen Xiao**: methodology; conceptualization. **Zibo Zhou**: writing—original draft; Visualization. **Yaoxue Xu**: resources; supervision. **Yonghao Pan**: writing—review & editing; funding acquisition. **Xiao Xia**: resources.

## CONFLICT OF INTEREST STATEMENT

The authors declare no conflict of interest.

## ETHICS STATEMENT

This study involved the secondary analysis of publicly available, completely de‐identified datasets (AAPM Mayo Clinic LDCT and LoDoPaB‐CT). Therefore, institutional review board (IRB) approval and patient informed consent were not required for this study.

## Data Availability

The datasets analyzed during the current study are publicly available. The Mayo Clinic LDCT dataset originates from the AAPM Low Dose CT Grand Challenge. The LoDoPaB‐CT dataset is available via its official benchmark repository. The code and trained model weights used in this study will be made publicly available upon publication, subject to institutional and licensing constraints.

## References

[1] Mohammadinejad P , Mileto A , Yu L , et al. CT noise‐reduction methods for lower‐dose scanning: strengths and weaknesses of iterative reconstruction algorithms and new techniques. Radiographics. 2021;41(5):1493‐1508. doi:10.1148/rg.2021200196 34469209

[2] Sidky EY , Pan X . Image reconstruction in circular cone‐beam computed tomography by constrained, total‐variation minimization. Phys Med Biol. 2008;53(17):4777‐4807. doi:10.1088/0031-9155/53/17/021 18701771 PMC2630711

[3] Thibault JB , Sauer KD , Bouman CA , Hsieh J . A three‐dimensional statistical approach to improved image quality for multislice helical CT. Med Phys. 2007;34(11):4526‐4544. doi:10.1118/1.2789499 18072519

[4] Elbakri IA , Fessler JA . Statistical image reconstruction for polyenergetic X‐ray computed tomography. IEEE Trans Med Imaging. 2002;21(2):89‐99. doi:10.1109/42.993128 11929108

[5] Beister M , Kolditz D , Kalender WA . Iterative reconstruction methods in X‐ray CT. Phys Med. 2012;28(2):94‐108. doi:10.1016/j.ejmp.2012.01.003 22316498

[6] Buades A , Coll B , Morel JM . A non‐local algorithm for image denoising. In: Proceedings of the 2005 IEEE Computer Society Conference on Computer Vision and Pattern Recognition . 2005;2:60‐65. doi:10.1109/CVPR.2005.38

[7] Dabov K , Foi A , Katkovnik V , Egiazarian K . Image denoising by sparse 3‐D transform‐domain collaborative filtering. IEEE Trans Image Process. 2007;16(8):2080‐2095. doi:10.1109/TIP.2007.901238 17688213

[8] Wiener N . Extrapolation, Interpolation, and Smoothing of Stationary Time Series. MIT Press; 1949. doi:10.7551/mitpress/2946.001.0001

[9] Lim JS . Two‐Dimensional Signal and Image Processing. Prentice Hall; 1990.

[10] Rudin LI , Osher S , Fatemi E . Nonlinear total variation based noise removal algorithms. Phys D. 1992;60(1‐4):259‐268. doi:10.1016/0167-2789(92)90242-F

[11] Chambolle A . An algorithm for total variation minimization and applications. J Math Imaging Vis. 2004;20(1‐2):89‐97. doi:10.1023/B:JMIV.0000011325.36760.1e

[12] Chen H , Zhang Y , Zhang W , et al. Low‐dose CT via convolutional neural network. Biomed Opt Express. 2017;8(2):679‐694. doi:10.1364/BOE.8.000679 28270976 PMC5330597

[13] Chen H , Zhang Y , Kalra MK , et al. Low‐dose CT with a residual encoder‐decoder convolutional neural network. IEEE Trans Med Imaging. 2017;36(12):2524‐2535. doi:10.1109/TMI.2017.2715284 28622671 PMC5727581

[14] Goodfellow I , Pouget‐Abadie J , Mirza M , et al. Generative adversarial nets. In: Advances in Neural Information Processing Systems . 2014: 2672‐2680. https://papers.nips.cc/paper/5423‐generative‐adversarial‐nets

[15] Wolterink JM , Leiner T , Viergever MA , Išgum I . Generative adversarial networks for noise reduction in low‐dose CT. IEEE Trans Med Imaging. 2017;36(12):2536‐2545. doi:10.1109/TMI.2017.2708985 28574346

[16] Arjovsky M , Chintala S , Bottou L . Wasserstein GAN. In: Proceedings of the 34th International Conference on Machine Learning (ICML) . 2017:214‐223. https://proceedings.mlr.press/v70/arjovsky17a.html

[17] Gulrajani I , Ahmed F , Arjovsky M , Dumoulin V , Courville A . Improved training of Wasserstein GANs. In: Advances in Neural Information Processing Systems . 2017:5767‐5777. https://papers.nips.cc/paper/7159‐improved‐training‐of‐wasserstein‐gans

[18] Johnson J , Alahi A , Fei‐Fei L . Perceptual losses for real‐time style transfer and super‐resolution. In: Computer Vision–ECCV 2016 . Springer; 2016:694‐711. doi:10.1007/978-3-319-46475-6_43

[19] Simonyan K , Zisserman A . Very deep convolutional networks for large‐scale image recognition. In: Proceedings of the 3rd International Conference on Learning Representations (ICLR) . 2015.

[20] Yang Q , Yan P , Zhang Y , et al. Low‐dose CT image denoising using a generative adversarial network with Wasserstein distance and perceptual loss. IEEE Trans Med Imaging. 2018;37(6):1348‐1357. doi:10.1109/TMI.2017.2783592 29870364 PMC6021013

[21] Wang Z , Cun X , Bao J , Zhou W , Liu J , Li H . Uformer: a general U‐shaped transformer for image restoration. In: Proceedings of the IEEE/CVF Conference on Computer Vision and Pattern Recognition . 2022:17683‐17693. doi:10.1109/CVPR52688.2022.01716

[22] Wang D , Fan F , Wu Z , Liu R , Wang F , Yu H . CTformer: convolution‐free Token2Token dilated vision transformer for low‐dose CT denoising. Phys Med Biol. 2023;68(6):065012. doi:10.1088/1361-6560/acbf11 36854190

[23] Burt PJ , Adelson EH . The Laplacian pyramid as a compact image code. IEEE Trans Commun. 1983;31(4):532‐540. doi:10.1109/TCOM.1983.1095851

[24] Srivastava N , Hinton G , Krizhevsky A , Sutskever I , Salakhutdinov R . Dropout: a simple way to prevent neural networks from overfitting. J Mach Learn Res. 2014;15(1):1929‐1958. https://www.jmlr.org/papers/v15/srivastava14a.html

[25] Isola P , Zhu JY , Zhou T , Efros AA . Image‐to‐image translation with conditional adversarial networks. In: Proceedings of the IEEE Conference on Computer Vision and Pattern Recognition . 2017:1125‐1134. doi:10.1109/CVPR.2017.632

[26] Gu A , Mamba DT . Linear‐time sequence modeling with selective state spaces. arXiv:2312.00752 . Preprint posted online December 1, 2023. https://arxiv.org/abs/2312.00752

[27] Chen Y , Fan H , Xu B , et al. Drop an octave: reducing spatial redundancy in convolutional neural networks with octave convolution. In: Proceedings of the IEEE/CVF International Conference on Computer Vision . 2019:3435‐3444. doi:10.1109/ICCV.2019.00353

[28] Katta S , Singh P , Garg D , Diwakar M . A hybrid approach for CT image noise reduction combining method noise‐CNN and shearlet transform. Biomed Pharmacol J. 2024;17(3):1875‐1898. doi:10.13005/bpj/2991

[29] Aneesh C , Saumik G , Varun KVV , Afnaan K , Singh T , Mandal A . CT image denoising using autoencoder and generative adversarial networks. In: 024 IEEE Recent Advances in Intelligent Computational Systems (RAICS). IEEE; 2024:1‐6. doi:10.1109/RAICS61201.2024.10689745

[30] Sadia RT , Chen J , Zhang J . CT image denoising methods for image quality improvement and radiation dose reduction. J Appl Clin Med Phys. 2024;25(2):e14270. doi:10.1002/acm2.14270 38240466 PMC10860577

[31] Brenner DJ , Hall EJ . Computed tomography—an increasing source of radiation exposure. N Engl J Med. 2007;357(22):2277‐2284. doi:10.1056/NEJMra072149 18046031

[32] Kalra MK , Maher MM , Toth TL , et al. Strategies for CT radiation dose optimization. Radiology. 2004;230(3):619‐628. doi:10.1148/radiol.2303021726 14739312

[33] McCollough CH , Primak AN , Braun N , Kofler J , Yu L , Christner J . Strategies for reducing radiation dose in CT. Radiol Clin North Am. 2009;47(1):27‐40. doi:10.1016/j.rcl.2008.10.006 19195532 PMC2743386

[34] Hsieh J . Computed Tomography: Principles, Design, Artifacts, and Recent Advances. 3rd ed. SPIE Press; 2015. doi:10.1117/3.2197756

[35] Willemink MJ , Noël PB . The evolution of image reconstruction for CT from filtered back projection to deep learning. Eur Radiol. 2019;29(5):2185‐2195. doi:10.1007/s00330-018-5810-4 30377791 PMC6443602

[36] Samei E , Bakalyar D , Boedeker KL , et al. Performance evaluation of computed tomography systems: summary of AAPM Task Group 233. Med Phys. 2019;46(11):e735‐e756. doi:10.1002/mp.13763 31408540

[37] Zhang K , Zuo W , Chen Y , Meng D , Zhang L . Beyond a Gaussian denoiser: residual learning of deep CNN for image denoising. IEEE Trans Image Process. 2017;26(7):3142‐3155. doi:10.1109/TIP.2017.2662206 28166495

[38] Lehtinen J , Munkberg J , Hasselgren J , et al. Noise2Noise: learning image restoration without clean data. In: Proceedings of the 35th International Conference on Machine Learning . 2018:2965‐2974. https://proceedings.mlr.press/v80/lehtinen18a.html

[39] Krull A , Buchholz TO , Jug F . Noise2Void: learning denoising from single noisy images. In: Proceedings of the IEEE/CVF Conference on Computer Vision and Pattern Recognition . 2019:2129‐2137. doi:10.1109/CVPR.2019.00223

[40] Ronneberger O , Fischer P , Brox T . U‐Net: convolutional networks for biomedical image segmentation. In: Medical Image Computing and Computer‐Assisted Intervention (MICCAI) . 2015:234‐241. doi:10.1007/978-3-319-24574-4_28

[41] He K , Zhang X , Ren S , Sun J . Deep residual learning for image recognition. In: Proceedings of the IEEE Conference on Computer Vision and Pattern Recognition . 2016:770‐778. doi:10.1109/CVPR.2016.90

[42] Shan H , Zhang Y , Yang Q , et al. 3‐D convolutional encoder‐decoder network for low‐dose CT via transfer learning from a 2‐D trained network. IEEE Trans Med Imaging. 2018;37(6):1522‐1534. doi:10.1109/TMI.2018.2832217 29870379 PMC6022756

[43] Huang Z , Zhang J , Zhang Y , Shan H . DU‐GAN: generative adversarial networks with dual‐domain U‐Net‐based discriminators for low‐dose CT denoising. IEEE Trans Instrum Meas. 2022;71:1‐12. doi:10.1109/TIM.2021.3128703

[44] Liu Z , Lin Y , Cao Y , et al. Swin transformer: hierarchical vision transformer using shifted windows. In: Proceedings of the IEEE/CVF International Conference on Computer Vision . 2021:9992‐10002. doi:10.1109/ICCV48922.2021.00986

[45] Wang Q , Wu B , Zhu P , Li P , Zuo W , Hu Q . ECA‐Net: efficient channel attention for deep convolutional neural networks. In: Proceedings of the IEEE/CVF Conference on Computer Vision and Pattern Recognition . 2020:11531‐11539. doi:10.1109/CVPR42600.2020.01155

[46] Wang Z , Bovik AC , Sheikh HR , Simoncelli EP . Image quality assessment: from error visibility to structural similarity. IEEE Trans Image Process. 2004;13(4):600‐612. doi:10.1109/TIP.2003.819861 15376593

[47] McCollough CH , Bartley AC , Carter RE , et al. Low‐dose CT for the detection and classification of metastatic liver lesions: results of the 2016 low dose CT grand challenge. Med Phys. 2017;44(10):e339‐e352. doi:10.1002/mp.12345 29027235 PMC5656004

[48] Leuschner J , Schmidt M , Baguer DO , Maass P . LoDoPaB‐CT, a benchmark dataset for low‐dose computed tomography reconstruction. Sci Data. 2021;8(1):109. doi:10.1038/s41597-021-00893-z 33863917 PMC8052416

[49] Kingma DP , Ba J . Adam: a method for stochastic optimization. In: Proceedings of the 3rd International Conference on Learning Representations (ICLR) . 2015.

[50] Loshchilov I , Hutter F . SGDR: stochastic gradient descent with warm restarts. In: International Conference on Learning Representations (ICLR) . 2017. https://openreview.net/forum?id=Skq89Scxx

[51] Loshchilov I , Hutter F . Decoupled weight decay regularization. In: International Conference on Learning Representations (ICLR) . 2019. https://openreview.net/forum?id=Bkg6RiCqY7

[52] Polyak BT , Juditsky AB . Acceleration of stochastic approximation by averaging. SIAM J Control Optim. 1992;30(4):838‐855. doi:10.1137/0330046

[53] Fan M , Thayib T , McCollough C , Yu L . Accurate and efficient measurement of channelized hotelling observer‐based low‐contrast detectability on the ACR CT accreditation phantom. Med Phys. 2023;50(2):737‐749. doi:10.1002/mp.16068 36273393 10.1002/mp.16068 PMC9931649

